# Molecular Ageing of Alpha- and Beta-Synucleins: Protein Damage and Repair Mechanisms

**DOI:** 10.1371/journal.pone.0061442

**Published:** 2013-04-22

**Authors:** Vasanthy Vigneswara, Simon Cass, Declan Wayne, Edward L. Bolt, David E. Ray, Wayne G. Carter

**Affiliations:** 1 School of Biomedical Sciences, The University of Nottingham, Queen's Medical Centre, Nottingham, United Kingdom; 2 School of Graduate Entry Medicine and Health, University of Nottingham Medical School, Royal Derby Hospital, Derby, United Kingdom; Johns Hopkins University, United States of America

## Abstract

Abnormal α-synuclein aggregates are hallmarks of a number of neurodegenerative diseases. Alpha synuclein and β-synucleins are susceptible to post-translational modification as isoaspartate protein damage, which is regulated *in vivo* by the action of the repair enzyme protein L-isoaspartyl *O*-methyltransferase (PIMT). We aged *in vitro* native α-synuclein, the α-synuclein familial mutants A30P and A53T that give rise to Parkinsonian phenotypes, and β-synuclein, at physiological pH and temperature for a time course of up to 20 days. Resolution of native α-synuclein and β-synuclein by two dimensional techniques showed the accumulation of a number of post-translationally modified forms of both proteins. The levels of isoaspartate formed over the 20 day time course were quantified by exogenous methylation with PIMT using *S*-Adenosyl-L-[^3^H-*methyl*]methionine as a methyl donor, and liquid scintillation counting of liberated ^3^H-methanol. All α-synuclein proteins accumulated isoaspartate at ∼1% of molecules/day, ∼20 times faster than for β-synuclein. This disparity between rates of isoaspartate was confirmed by exogenous methylation of synucleins by PIMT, protein resolution by one-dimensional denaturing gel electrophoresis, and visualisation of ^3^H-methyl esters by autoradiography. Protein silver staining and autoradiography also revealed that α-synucleins accumulated stable oligomers that were resistant to denaturing conditions, and which also contained isoaspartate. Co-incubation of approximately equimolar β-synuclein with α-synuclein resulted in a significant reduction of isoaspartate formed in all α-synucleins after 20 days of ageing. Co-incubated α- and β-synucleins, or α, or β synucleins alone, were resolved by non-denaturing size exclusion chromatography and all formed oligomers of ∼57.5 kDa; consistent with tetramerization. Direct association of α-synuclein with β-synuclein in column fractions or from *in vitro* ageing co-incubations was demonstrated by their co-immunoprecipitation. These results provide an insight into the molecular differences between α- and β-synucleins during ageing, and highlight the susceptibility of α-synuclein to protein damage, and the potential protective role of β-synuclein.

## Introduction

The family of cytoplasmic synuclein proteins that comprises α-synuclein, β-synuclein, and γ-synuclein are thought to function in synaptic vesicle release and transmission, and neuronal plasticity. Alpha and β-synucleins are highly homologous proteins (62% identical) that are co-localised within presynaptic nerve terminals in the central nervous system, whereas γ-synuclein is primarily expressed in the peripheral nervous system [1]–[3].

Abnormal α-synuclein accumulations are hallmarks and presumed pathogenic events in a number of age-related diseases, collectively termed synucleopathies, and include Parkinson's disease (PD), Alzheimer's disease (AD), dementia with Lewy bodies (DLB), and multiple system atrophy (MSA) [3]. Native α-synuclein is an unfolded protein, but can undergo aggregation and fibril formation in a complex process that can be influenced by the local and external environment. Whether α-synuclein aggregates contribute to disease pathology, and/or induce cellular changes that trigger cellular toxicity and cell death is still under investigation, but a causative role of abnormal α-synuclein function is underscored by rare autosomal dominant mutants of α-synuclein, or α-synuclein gene multiplication, which give rise to Parkinsonian phenotypes [4]–[7]. Additionally, experimental animal models such as transgenic mice that express α-synuclein develop a Parkinsonian movement disorder and exhibit loss of dopaminergic neurons, a characteristic feature of PD [8].

One of the strategies employed to combat or curb disease pathology has been the development of therapies directed toward reducing α-synuclein aggregation and/or fibril formation [9], [10]. An example of this has been the co-incubation of β-synuclein with α-synuclein, since these two proteins directly bind one another, and their association reduces α-synuclein aggregation/fibril formation, and ameliorates α-synuclein-induced neurodegenerative manifestations [9]–[14].

**Figure 1:**
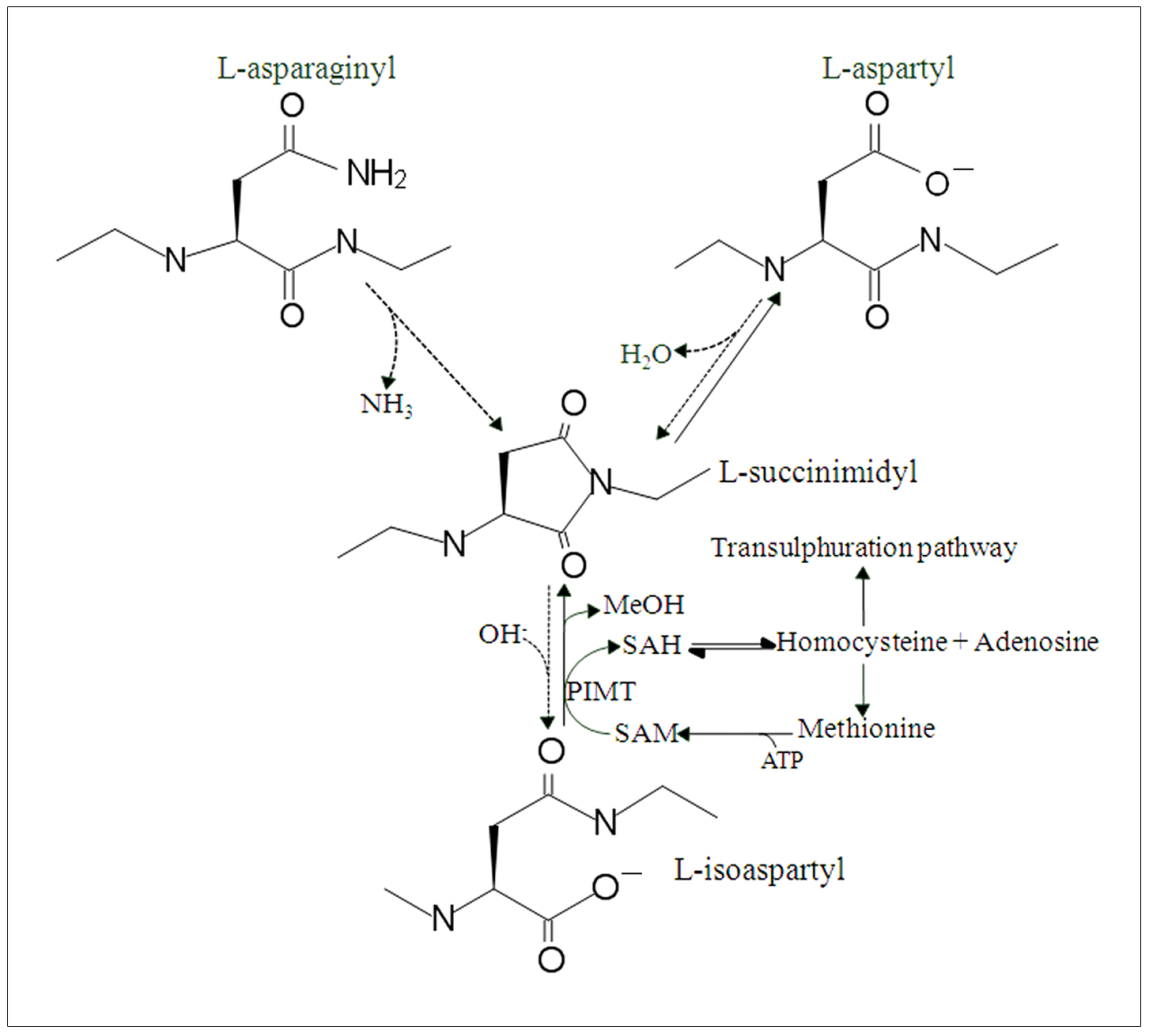
Isoaspartate protein damage formation and PIMT-mediated protein repair. Within a peptide backbone, non-enzymatic deamidation of an asparagine-Xaa linkage or dehydration of an aspartic acid-Xaa linkage can give rise to a five-carbon succinimide (indicated by the dashed arrows). Succinimide hydrolysis (OH^−^) yields an isoaspartate residue (major product, indicated by the dashed arrow), or an aspartic acid (minor product, indicated by the right solid arrow). PIMT utilises SAM to carboxyl-methylate the isoaspartate forming a methyl ester that readily hydrolyses under physiological conditions to liberate methanol and reform the five-carbon succinimide (lower solid arrow). Subsequent succinimide hydrolysis will again yield isoaspartate or ‘repaired’ aspartate products (right solid arrow). PIMT activity is influenced by the levels of SAM and SAH, which are likewise governed by the enzyme and metabolite levels of the methionine metabolic pathway.

The functional activity and aggregation potential of α-synuclein may be influenced by post-translational modifications that include phosphorylation, ubiquitination, and protein truncation [15]. Previously, our proteomic studies also identified α-synuclein and β-synuclein as substrates of methylation by the protein repair enzyme, protein L-isoaspartate *O*-methyltransferase (PIMT) [16]. PIMT is a ubiquitously expressed methyltransferase which methylates isoaspartic acid residues in cytosolic and nuclear proteins. Isoaspartate is a protein post-translational modification that arises as a result of the lability of asparagine and aspartic acid residues within peptide and protein chains. Non-enzymatic deamidation of an asparagine residue or dehydration of an aspartic acid residue can give rise to a five-carbon succinimide, whose subsequent hydrolysis can generate a return of an aspartic acid (∼30% of yield), or an isoaspartatic acid residue (∼70% of yield). Isoaspartate formation produces a ‘kink’ in a peptide chain since the peptide backbone now contains an extra methylene group and is maintained through the β-carbonyl group of the generating amino acid – refer to Figure 1. This alteration of protein structure may be concomitant with loss of protein function, so isoaspartate formation is considered a common and generally undesired form of protein damage [16]–[24].

PIMT utilises *S*-adenosylmethionine (SAM) as a methyl donor to carboxyl-methylate isoaspartate forming a methyl ester that readily hydrolyses under physiological conditions to liberate methanol, and reform the five-carbon succinimide. Succinimide hydrolysis will again yield aspartate and isoaspartate products. Hence successive rounds of PIMT methylation of isoaspartate, succinimide formation and then hydrolysis, will form aspartic acid residues within normal peptide linkages, and this has been shown to be concomitant with restoration of protein function - refer to Figure 1 [16]–[24].

The importance of functional activity of PIMT and repair of isoaspartate peptide linkages was illustrated by the production and analysis of PIMT knockout (*Pcmt1−/−*) mice [16], [25]–[31]. These mice accumulate isoaspartate protein damage in tissues that express PIMT, with the highest levels of isoaspartate damage detected within neuronal tissue proteins [16]. *Pcmt1−/−* mice display neuronal abnormalities that include aberrant synaptic neurotransmission, and most animals succumb to a terminal epileptic seizure by two months of age [16], [25]–[31].

The byproduct of PIMT methylation reactions is *S*-adenosylhomocysteine (SAH), which is a product inhibitor of many methyltransferases including PIMT [32]–[37]. The protein repair activity of PIMT is therefore susceptible to agents that impinge upon the methionine metabolic pathway and result in a change in the ratio of SAM∶SAH [32]–[37]. Agents that lower the SAM∶SAH ratio, for example, excessive alcohol consumption, folate deficiency, or inhibitors of SAH hydrolase, result in an inhibition of PIMT activity, and an increase in isoaspartate protein damage within cellular proteins [33]–[37].

**Figure 2:**
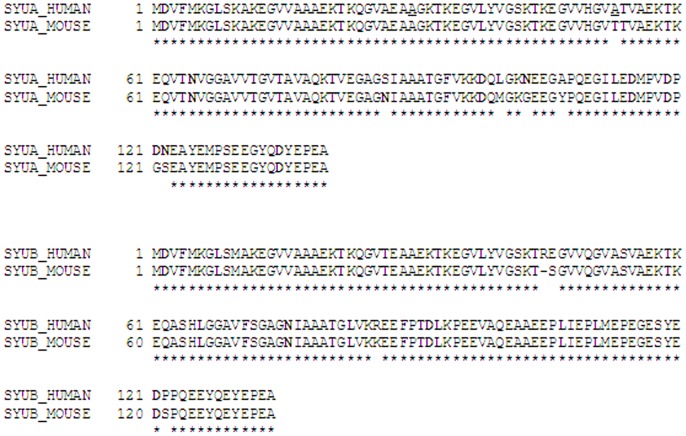
Amino acid sequence alignment of human and mouse α-synuclein, and human and mouse β-synuclein. Identical amino acids are marked with an asterisk. The positions of the human α-synuclein familial mutants A30P and A53T are underlined. Aspartic acid and asparagine residues that may give rise to isoaspartate formation are shown in bold.

Although α- and β-synucleins are highly homologous proteins, they may display unique levels of protein post-translational modifications which could influence their functional (patho)physiological activities. The formation of protein post-translational modification as isoaspartate has been modelled by ageing proteins *in vitro* at physiological pH and temperature, and quantitated by exogenous methylation with PIMT using ^3^H-SAM [22], [23], [38], [39]. Our previous proteomic study demonstrated that murine α-synuclein and murine β-synuclein form isoaspartate protein damage *in vivo* and are substrates of PIMT [16]. Human α- and human β-synucleins possess 95 and 97% sequence homology respectively to their murine counterparts (Figure 2). The aim of this study was to examine the formation of isoasparate protein damage after *in vitro* aging human α-synuclein, human β-synuclein, and the mutants of human α-synuclein, A30P and A53T that can trigger familial Parkionsonian phenotypes.

## Experimental Procedures (Materials and Methods)

Recombinant human α-synuclein (MW = 14460, product AG938), β-synuclein (MW = 14288, product AG946), A30P mutant α–synuclein (MW = 14486, product AG942), and A53T mutant α-synuclein (MW = 14490, product AG940) were purchased from Chemicon International, USA. Immobilised pH gradient (IPG) strips (pH 4–7, 7 cm length) were purchased from BioRad, with all isoelectric focussing performed using a BioRad Protean isoelectric focussing cell. NuPAGE Novex pre-cast gels (4–12% Bis-Tris gels for 1D SDS-PAGE and 4–12% Bis-Tris Zoom gels for 2D PAGE analysis), 2-(N-morpholino)ethanesulfonic acid (MES)-SDS running buffer, transfer buffer, SeeBlue Plus2 prestained gel standards, and Safe stain were all bought from the Invitrogen Corporation. All other SDS-PAGE reagents were purchased from Sigma. Isoquant isoaspartate detection kits were purchased from the Promega Corporation. *S*-Adenosyl-L-[^3^H-*methyl*]methionine (37 MBq/ml), destreak reagent, and PlusOne silver staining kits were purchased from Amersham Biosciences UK. Bovine recombinant PIMT provided with the Isoquant kit was used for exogenous isoaspartate methylation prior to protein separation using 1D or 2D-PAGE. Rabbit polyclonal antibody specific for α-synuclein (sc-7011-R) was purchased from Santa-Cruz, USA. Rabbit polyclonal antibody specific for β-synuclein (SA3405) was purchased from Enzo. Goat anti-rabbit immunoglobulins-horseradish peroxidase conjugated secondary antibody (P0448) was purchased from Dako, UK.

### Mice

Brain cytosolic proteins from *Pcmt1+/+* (wild-type) and *Pcmt1−/−* (PIMT knockout (KO)) mice were kindly provided by the laboratory of Professor Steven Clarke (University of California at Los Angeles). Cytosolic proteins were shipped on dry ice to the laboratory of WGC, and maintained at −80°C until required for analyses. The production and breeding conditions for generation of these mice have been reported previously [25], [28]. Mice were monitored by on-site veterinarians, with all protocols undertaken in strict accordance with the recommendations for the Care and Use of Laboratory Animals, and approval by the University of California at Los Angeles Institutional Animal Care and Use Committee, under approved protocol ARC #1993-109-62. The preparation of *Pcmt1−/−* brain cytosolic proteins were as described previously [40].

### *In vitro* aging of synucleins

Synucleins were dissolved in an *in vitro* aging buffer of 50 mM K-HEPES pH 7.4, 1 mM EDTA, 5% glycerol, 0.02% sodium azide to a concentration of 1 mg/ml (approximately 70 µM). Proteins were aged at 37°C for up to 20 days, with protein samples removed after 0, 2, 5, 9, and 20 days of *in vitro* ageing, immediately flash frozen, and stored at −80°C until required. These buffer conditions are similar to those that have been used in other *in vitro* ageing studies [22], [23], [38], [39].

### Two dimensional polyacrylamide gel electrophoresis (2D-PAGE)

Six hundred µg of *Pcmt1−/−* brain cytosolic extract was incubated either with or without 5 µg of 20 day *in vitro* aged α-synuclein or 20 day *in vitro* aged β-synuclein for resolution by 2D-PAGE and Coomassie protein staining. For autoradiography studies, 2D-PAGE was preceded by methylation of 5 µg of 20 day *in vitro* aged α-synuclein or 20 day *in vitro* aged β-synuclein in a buffer of 50 mM K-MES, pH 6.2, containing 20 µM ^3^H-SAM (8250 dpm/pmol final specific activity) in a final volume of 20 µl. Methylation was initiated by the addition of 5 µl of exogenous recombinant bovine PIMT (2.4 µM final concentration), and continued for 30 minutes at 30°C. This exogenous methylation of the l-isoaspartate synuclein proteins resulted in the formation of ^3^H-methyl esters. Experimental conditions were then employed to preserve base-labile ^3^H-methyl esters to enable isoaspartate detection via autoradiography [16]. Proteins for 2D-PAGE and Coomassie staining or autoradiography were processed similarly. Proteins were precipitated by incubation with 700 µl of protein precipitation solution (acetone∶diethyl ether (2∶1 (v/v)), and samples placed on ice for 15 minutes. Precipitated protein was collected by centrifugation at 5000 rpm for 3 minutes, washed three times with ether∶industrial methylated spirit∶water (10∶7∶2 (v/v/v)), air dried, and then proteins dissolved in a rehydration buffer (9.8 M urea, 2% (w/v) CHAPS, 0.5% IPG buffer) containing 12.5 µl/ml of the anti-oxidant destreak reagent, at room temperature for 1 hour. Solubilised protein was actively rehydrated into 7 cm pH 4–7 IPG isoelectric focusing strips for 16 hours at 50 V, and then focussed for 16 hours according to the manufacturer's guidelines. Focussed strips were washed in an equilibration buffer of 0.375 M Tris/HCl pH 6.8, 6 M urea, 2% SDS, 20% glycerol, containing 2% (w/v) dithiothreitol (DTT) for 10 minutes, and then similarly washed with the same buffer in which 2.5% (w/v) iodoacetamide replaced the DTT. After this reduction and alkylation, strips were equilibrated in MES running buffer before application to the top of NuPage Zoom gels, and protein separation by SDS-PAGE [16]. Proteins resolved by 2D-PAGE were fixed by washing in 50% methanol, 10% acetic acid, and then stained with colloidal Coomassie blue according to the manufacturer's recommendations. 2D-PAGE co-migrational experiments containing *Pcmt1−/−* brain cytosolic proteins and α-synuclein or β-synuclein were performed 2–3 times with each synuclein. Two-dimensional PAGE resolution of *Pcmt1+/+* or *Pcmt1−/−* brain cytosolic proteins have been performed greater than 5 times with each genotype, with migrational positions of α-synuclein and β-synuclein previously validated [16].

### Quantitation of isoaspartate levels in synucleins by a methanol diffusion assay

Isoaspartate levels within synuclein proteins were quantified using an Isoquant kit by exogenous methylation with PIMT in a final volume of 50 µl utilising 20 µM ^3^H-SAM (1 µCi/reaction, 2220 dpm/pmol final specific activity), in a buffer of 100 mM sodium phosphate pH 6.8, containing 1 mM EGTA, 0.16% Triton X-100, and 0.004% sodium azide (final concentrations) for 30 minutes at 30°C. Methylation was terminated and the methyl esters hydrolyzed by the addition of an equivalent volume of CAPS (pH 10), 5% SDS, 2.2% methanol, 0.1% m-cresol purple, and the samples retained on ice for 10 minutes. Fifty microlitres from each of the samples was then spotted onto a sponge inserted into the cap of a scintillation vial. The samples were incubated for 60 min at 40°C to volatilize ^3^H-methanol from the methylated proteins into 10 ml of scintillation fluid, which was then counted for radioactivity. For each time point, one and a quarter micrograms of aged synucleins were assayed in duplicate from which an average was taken, and methylation blank subtracted. Fifty picomoles of isoaspartate-containing delta sleep-inducing peptide were similarly methylated to provide a reference level of isoaspartate methylation [16]. Levels of isoaspartate were analysed by one way analysis of variance (ANOVA) with Bonferroni post-test using Prism software. A *p* value of <0.05 was regarded as statistically significant. All data points for isoaspartate quantitation are representative of a mean and SEM from 3–5 independent experiments.

### One dimensional polyacrylamide gel electrophoresis (1D-PAGE)

Synuclein proteins were methylated by exogenous PIMT using ^3^H-SAM as described above for 2D-PAGE, but methylation terminated by the addition of a quarter of a volume of 5× concentrated reducing solution (10% SDS and 500 mM DTT) and the samples heated for 10 minutes at 50°C. One quarter of a volume of 5× Laemmli sample buffer was added (250 mM Tris/HCl, pH 6.8, 40% (v/v) glycerol, 5% (w/v) SDS, 0.005% (w/v) bromophenol blue) and synuclein proteins (2.5 µg/gel lane) resolved on 4–12% Bis/Tris gels for 2 hours at 125 V using MES running buffer (pH 7.3) run in an X Cell surelock gel tank (Invitrogen). Gel resolved synuclein proteins were stained with silver and then photographed using a Fugi E900 digital camera. Alternatively, gel separated synucleins were transferred at 80 V for 2 hours to a polyvinylidene difluoride (PVDF) membrane for either autoradiography or Western blotting.

### Autoradiography

Approximately 0.25 µl of 100 KBq ^14^C-amino acid mixture (1.85 MBq/ml, Amersham) was spotted onto the PVDF membrane at the positions of the molecular weight markers to enable their visualisation during autoradiography. Autoradiography was performed using a microchannel plate (MCP) autoradiographic imager. These novel devices are able to produce real-time digital autoradiographic images, from a detection threshold of 6 dpm/mm^2^, and an intrinsic background noise of ∼5×10^−6^ counts/second per pixel measurement [41]. This low signal threshold, and linearity over six orders of signal magnitude ably suit the detection and quantitation of relatively low energy β-particle (tritium) protein radiolabelling [41]. For proteins resolved by 1D-PAGE, α-synucleins were autoradiographed for 24 hours, and β-synuclein for 48 hours. For 2D-PAGE, PVDF membranes were autoradiographed for 48 hours.

### Western (immuno) blotting

PVDF membranes were stained with Coomassie blue Safe stain for 30 minutes, and then air-dried. Membranes were destained with 50% methanol, 10% acetic acid and then washed in phosphate buffered saline (PBS) containing 0.05% Tween-20 (PBS-T). Membranes were blocked in the same buffer containing 5% (w/v) milk fat, and then probed with the following antibodies in blocking buffer overnight at 4°C: rabbit polyclonal antibody specific for α-synuclein at a 1∶250 dilution, or rabbit polyclonal antibody specific for β-synuclein at a 1∶250 dilution (Enzo). Blots were washed, and then incubated with a goat anti-rabbit immunoglobulins-horseradish peroxidase-conjugated secondary antibody at 1∶1000 dilution for 1 hour at room temperature. Blots were similarly washed and then synuclein localisation visualised using enhanced chemiluminescence (ECL) (Pierce). Light generated from ECL was either captured on CL-Xposure X-ray film (Pierce), or using a closed circuit device (ChemiDoc, BioRad). X-ray films were scanned on an Epson stylus XS400 flatbed scanner.

### Matrix assisted laser-desorption ionisation-time of flight (MALDI-TOF) mass spectrometry

Silver stained proteins resolved by 1D PAGE were excised from gels and transferred to a 96-well plate using an automated MassPrep robotic system (ProteomeWorks, BioRad). After protein trypsinolysis, MALDI-TOF mass spectrometry was performed using a Micromass MALDI (Waters, UK) as described in a previous publication [42]. A number of intact singularly charged peptides in the mass range of 800–3000 Da were identified, and the masses applied to a search algorithm (MASCOT peptide mass fingerprint) to screen protein databases, such as SwissProt for peptide mass matches to enable protein identification.

### Column chromatography

Size exclusion chromatography was performed using a Superose 12 (GE Healthcare) column run on an AKTA FPLC system (Amersham Phamacia). Proteins were separated in a buffer of 50 mM Tris/HCl pH 7.4, 100 mM NaCl, 1 mM EDTA and 5% (v/v) glycerol at a flow rate of 0.5 ml/min. Protein samples (20–50 µg/run in 200 µl final volume) were resolved and 0.25 ml fractions collected. The approximate molecular weight of eluted synuclein proteins were estimated from comparison of their elution volumes with those of proteins of known molecular weight (gel filtration standards, Biorad: thyroglobulin (670 kDa), γ-globulin (158 kDa), ovalbumin (44 kDa), myoglobin (17 kDa), and vitamin B12 (1.35 kDa). Chromatography was performed at least once on all α, β, and co-incubation time points, with representative chromatograms displayed in Figures. Peak column fractions were concentrated using Microcon concentrators (3 kDa cut-off, Fisher), and proteins resolved by 1D SDS-PAGE. Proteins were stained with colloidal Coomassie blue (Invitrogen) and Western blotted as described above.

### Immunoprecipitation (IP) analyses

Approximately 20 µg of β-synuclein from *in vitro* aged reactions, or from peak column fractions from Superose 12 chromatography were precleared at 4°C for 1 hour, and then β-synuclein immunoprecipitated by the addition of 2 µg of anti-β-synuclein antibody or control rabbit serum (Dako), and end-over-end rotation on a carousel for 16 hours at 4°C. Immunoprecipitation was similarly performed on co-incubation mixtures of α- and β-synucleins. Immune complexes were captured by the addition of protein-A-agarose for 2 hours at 4°C, and then washed four times with a wash buffer of 20 mM Tris/HCl pH 7.5, 150 mM NaCl, 1% (v/v) Triton X-100. Protein was dissociated from the antibody by incubation in 20 µl of Laemmli sample buffer (Invitrogen) and heating at 70°C for 10 minutes. Proteins were resolved by 1D SDS-PAGE, transferred to a PVDF membrane, and then blotted for α- or β-synuclein as described in Figure legends.

## Results

**Figure 3:**
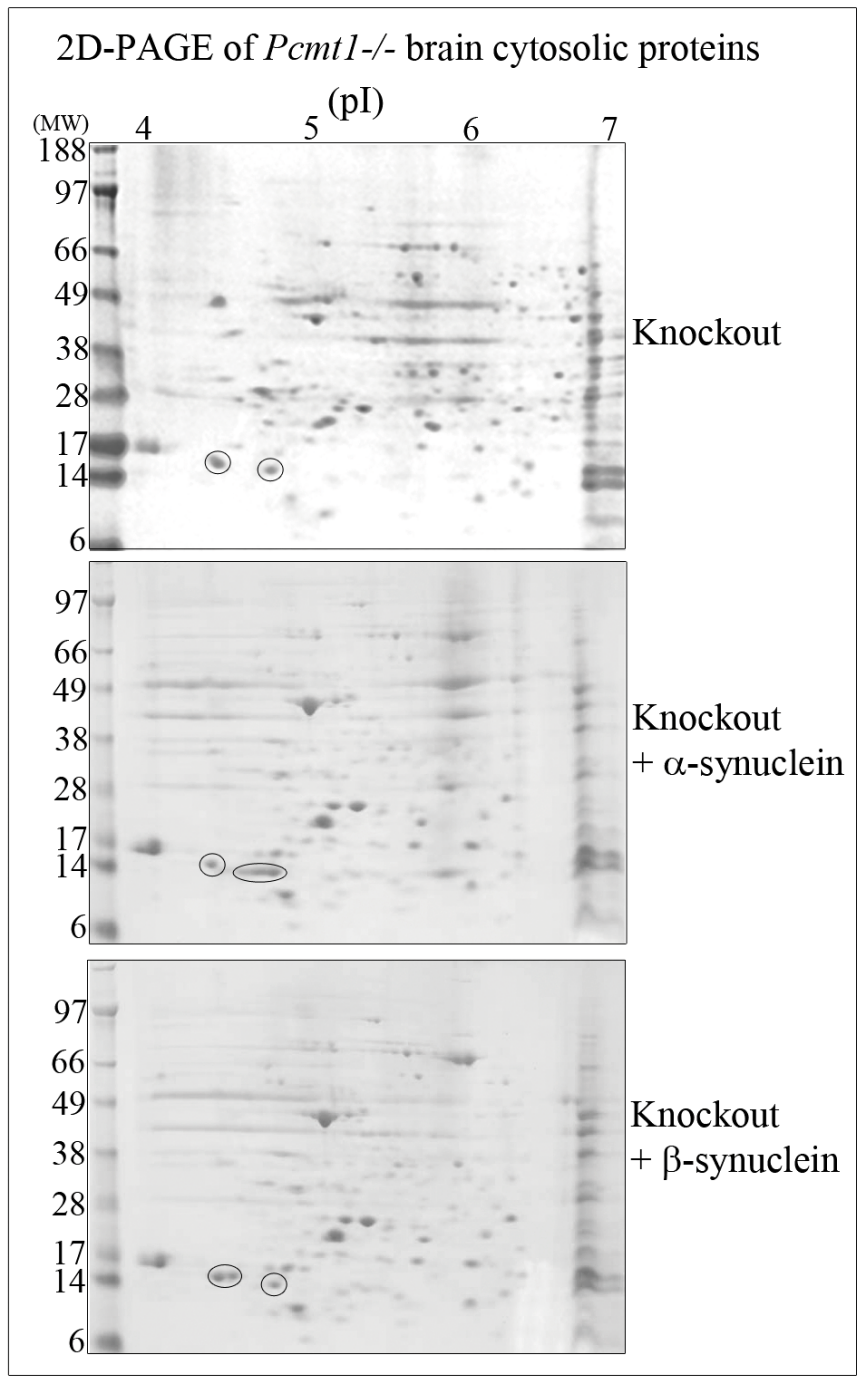
2D-PAGE of *Pcmt1−/−* mice brain cytosolic proteins. *Pcmt1−/−* brain cytosolic proteins were resolved by 2D-PAGE and proteins stained with colloidal Coomassie blue (upper panel). *Pcmt1−/−* brain cytosolic proteins co-incubated with 5 µg of α-synuclein (middle panel) or 5 µg of β-synuclein (lower panel) prior to co-migrational 2D-PAGE. Protein spots identified as α-synuclein (right spot, ringed) and β-synuclein (left spot, ringed) were increased in their protein staining intensity with co-migrational analyses.

**Figure 4:**
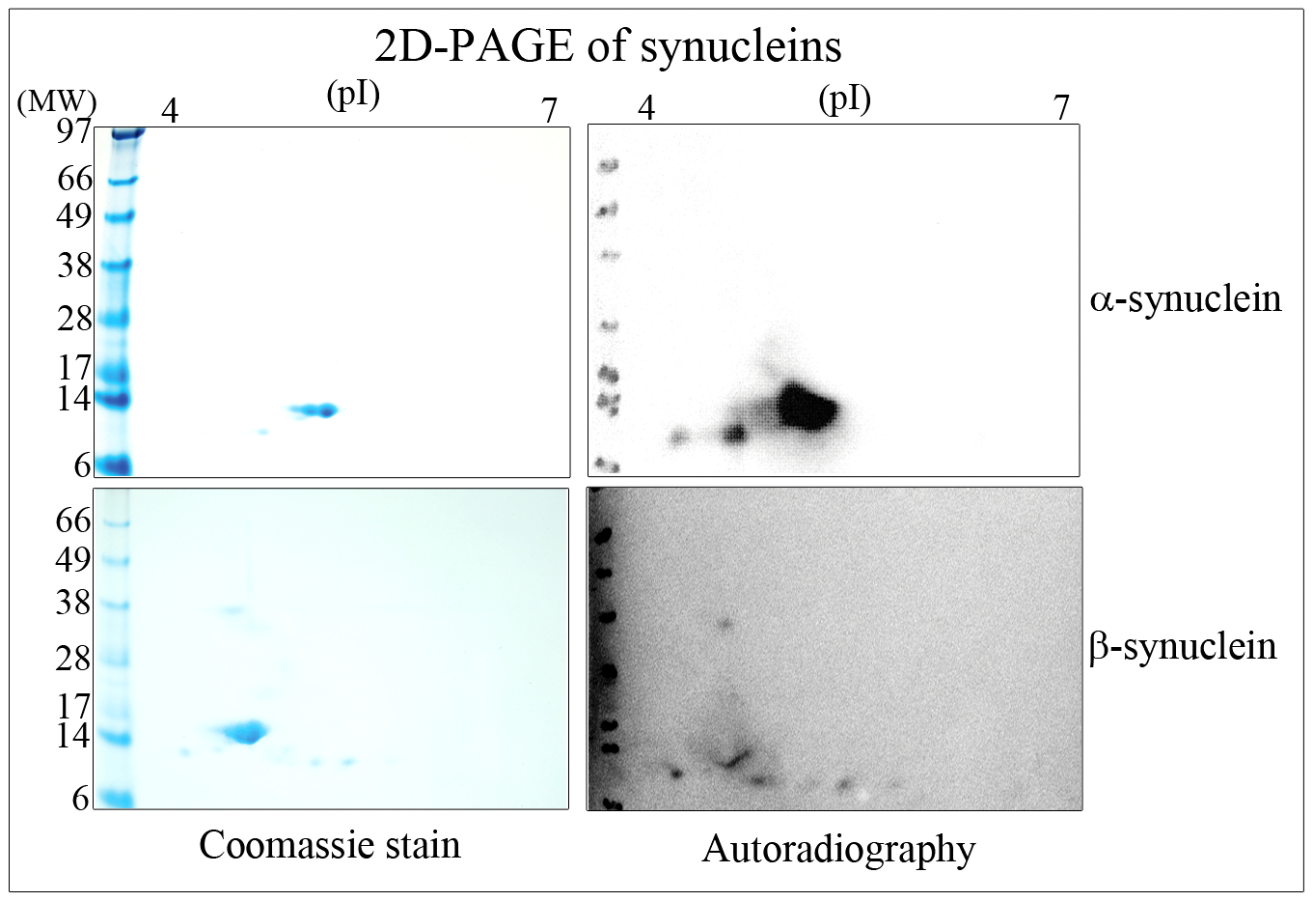
2D-PAGE of *in vitro* aged synucleins. Five µg of 20 day *in vitro* aged α-synuclein (upper panels) or 5 µg of 20 day *in vitro* aged β-synuclein (lower panels) were methylated with ^3^H-SAM using exogenous PIMT, and proteins resolved by 2D-PAGE. Proteins were stained with Coomassie (left panels), and methylation of isoaspartate visualised by autoradiography (right panels).

After *in vitro* aging at physiological pH and temperature for 20 days, recombinant human synucleins displayed similar protein separation character to that of their corresponding murine proteins from *Pcmt1−/−* brains, when resolved by co-migrational 2D-PAGE – Figure 3. Twenty day *in vitro* aged α-synuclein alone was resolved by 2D-PAGE into two major Coomassie stained proteins of pI ∼4.6–4.7, and two minor protein spots of approximate pI 4.2 and 4.4 and molecular weight ∼10 kDa (Figure 4, upper left panel). Two-dimensional PAGE resolution of 20 day *in vitro* aged β-synuclein alone typically showed one broad Coomassie stained protein at a pI of ∼4.4, and a number of other minor stained protein spots in the pI range of 4.2–4.8 and molecular weight of ∼10 kDa (Figure 4, lower left panel). In addition, α-synuclein and β-synuclein were methylated with ^3^H-SAM using exogenous PIMT, and then resolved by 2D-PAGE and protein radiolabelling of isoaspartate, as ^3^H-methyl esters, visualised by autoradiography. Noteworthy was that aged α-synuclein had accumulated significantly more isoaspartate than aged β-synuclein, as evidenced by the relatively higher autoradiographic signal – Figure 4, right panels. Both major Coomassie stained protein forms of α-synuclein contained isoaspartate peptide linkages, and isoaspartate was also localised to the minor lower molecular weight protein spots. The single prominent Coomassie stained β-synuclein protein only accumulated low levels of isoaspartate, as did the additional minor low molecular weight forms of this protein, and the ∼38 kDa spot that arose from incomplete 2D-PAGE resolution of β-synuclein protein – Figure 4, right panels.

**Figure 5:**
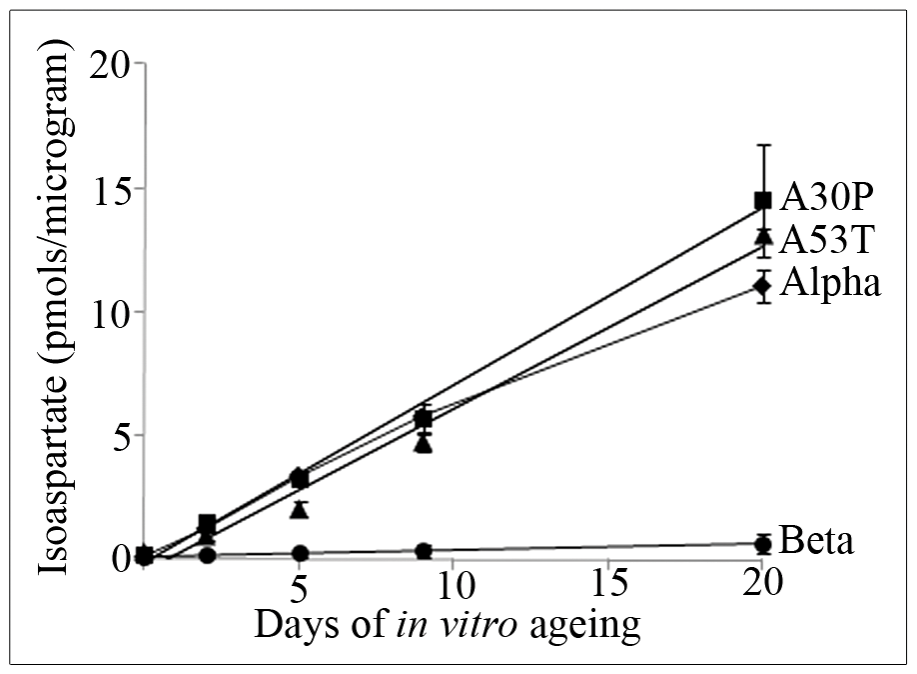
Quantitation of isoaspartate protein damage after *in vitro* ageing of synucleins. Native α-synuclein, β-synuclein, A30P mutant α-synuclein, or A53T mutant α-synuclein were *in vitro* aged over a time course of 20 days. Two and a half µg of protein was removed after 0, 2, 5, 9, and 20 days of *in vitro* ageing and the level of isoaspartate formation quantified by a methanol diffusion assay.

**Figure 6:**
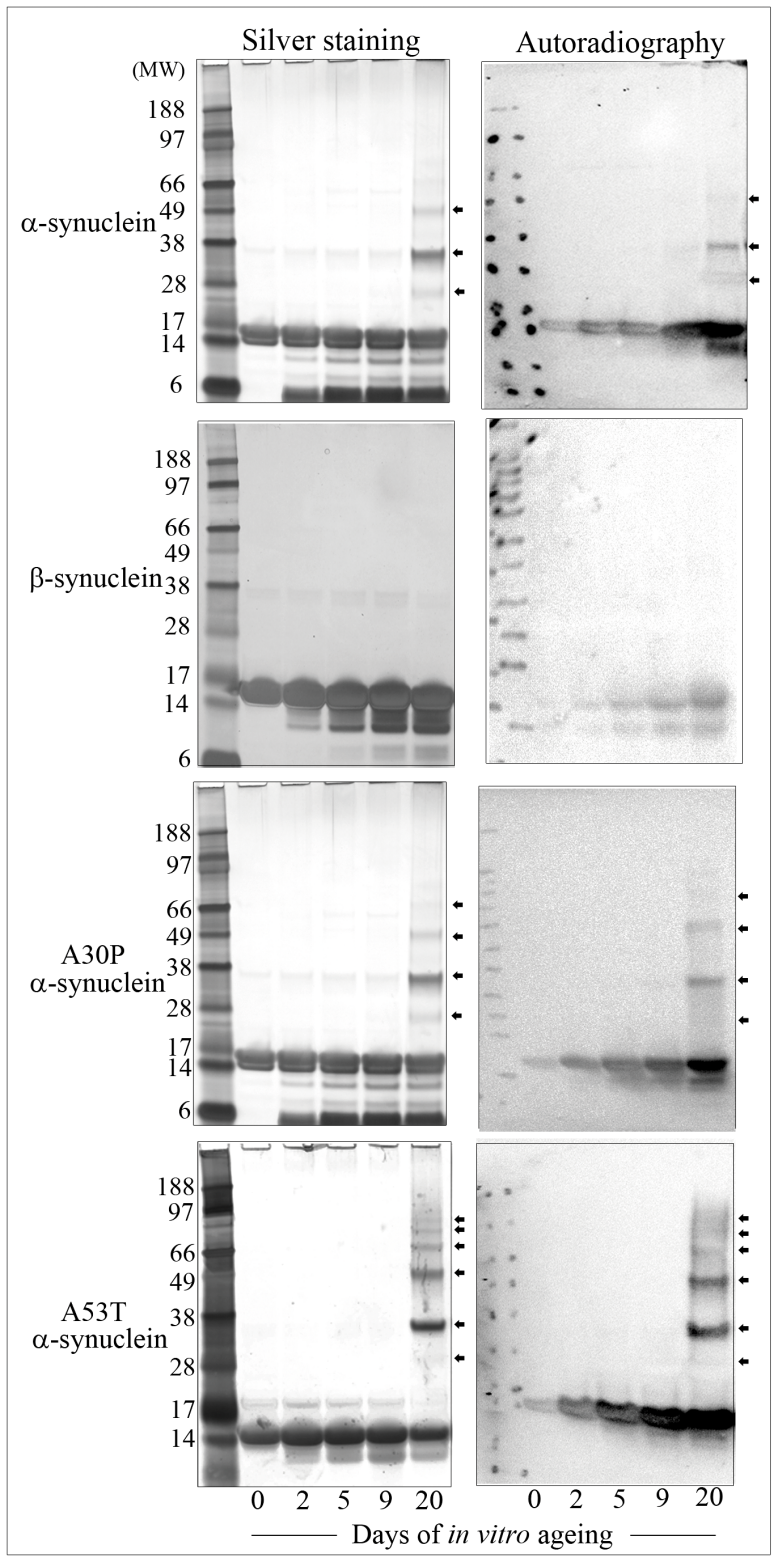
1D-PAGE resolution of *in vitro* aged and methylated synucleins, and methyl ester autoradiography. Synuclein proteins were methylated with ^3^H-SAM using exogenous PIMT, resolved by 1D PAGE and proteins stained with silver (left panels), or proteins transferred to PVDF and the level of isoaspartate methylation visualised by autoradiography (right panels). Wild-type and mutant α-synuclein proteins formed stable protein oligomers that contained isoaspartate peptide linkages (marked with arrowheads).

To investigate this disparity between the levels of isoaspartate that accumulated within aged α-synuclein compared to aged β-synuclein, we *in vitro* aged both proteins and removed samples for analysis after 0, 2, 5, 9, and 20 days of ageing. Additionally, we examined the formation of isoaspartate over the same time course for the autosomal dominant α-synuclein mutants A30P and A53T; mutant proteins that trigger familial PD. Levels of isoaspartate were quantified by exogenous methylation with ^3^H-SAM using PIMT, and liquid scintillation counting of ^3^H-methanol in a methanol diffusion assay. All α-synuclein proteins accumulated isoaspartate at a rate of ∼1% of molecules per day, whereas with β-synuclein this was reduced to ∼0.05% per day – Figure 5. This differential accumulation of isoaspartate between the α-synucleins and β-synuclein was further validated by isoaspartate methylation with ^3^H-SAM using exogenous PIMT, and then protein resolution of the ^3^H-methyl esters by 1D PAGE and their visualisation using digital autoradiography – Figure 6.

In addition, one dimensional PAGE and autoradiography revealed that native α-synuclein, and the familial mutant forms of α-synuclein; A30P and A53T, formed protein oligomers after *in vitro* ageing that were resistant to the denaturing conditions of SDS-PAGE. That these oligomers were α-synuclein protein was validated by both Western blotting with a specific anti-α-synuclein antibody, and excision of each protein band and mass spectrometry peptide mass fingerprinting. A sample Western blot and peptide mass fingerprint has been included as Supplementary (supporting) data. In contrast to α-synucleins, β-synuclein did not form these SDS-PAGE resistant oligomers over this 20 day time course.

**Figure 7:**
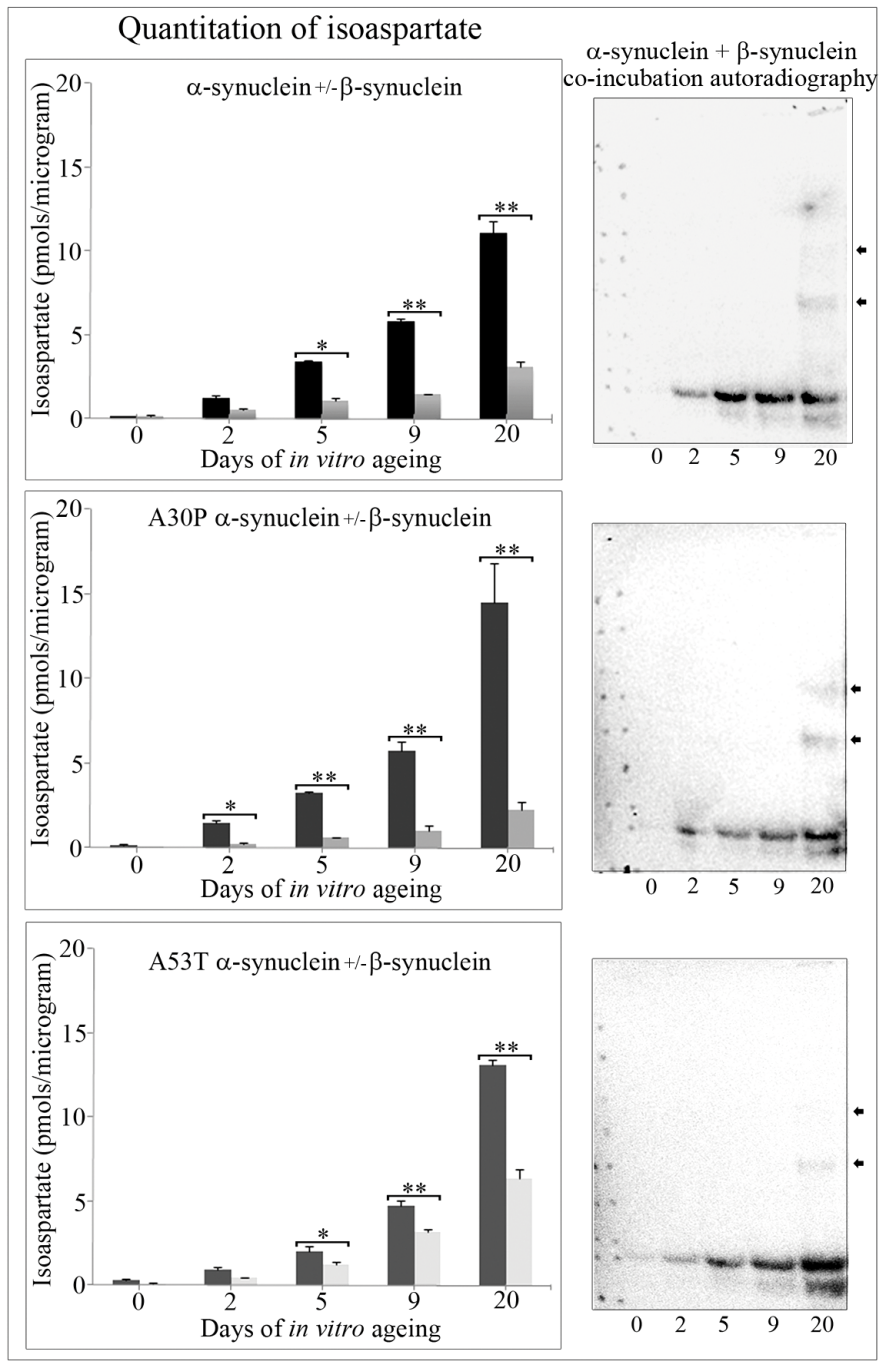
Quantitation of isoaspartate formation during *in vitro* ageing of α-synuclein either with or without co-incubation with β-synuclein, and co-incubation autoradiography. Alpha-synuclein, A30P mutant α-synuclein, or A53T mutant α-synuclein were *in vitro* aged either with or without β-synuclein over a time course of up to 20 days. Two and a half µg of protein was removed after 0, 2, 5, 9, and 20 days of *in vitro* ageing and the level of isoaspartate formation quantified by a methanol diffusion assay (left panels). Black filled columns correspond to α-synuclein isoaspartate values, grey filled columns correspond to isoaspartate levels from α-synuclein co-incubated with β-synuclein. Co-incubation of β-synuclein with α-synucleins significantly reduced total isoaspartate formed during the *in vitro* ageing time course. Significance is marked with an asterisk: *p<0.01; **p<0.001. Synuclein proteins from co-incubation experiments were methylated with ^3^H-SAM using exogenous PIMT, resolved by 1D PAGE, and isoaspartate methylation visualised by autoradiography (right panels). Stable α-synuclein protein oligomers that contained isoaspartate peptide linkages are marked with arrowheads.

A number of independent studies have suggested a neuroprotective role of β-synuclein due to its ability to associate with α-synuclein and reduce α-synuclein aggregation/fibril formation, and ameliorate α-synuclein-induced neurodegenerative manifestations [9]–[14], [43]. To investigate the influence of β-synuclein upon the formation of isoaspartate damage within α-synucleins, we co-incubated approximately equimolar β-synuclein with each α-synuclein, and quantified the rate of formation of isoaspartate over a time course of up to 20 days of *in vitro* ageing. At intervals of 0, 2, 5, 9, and 20 days, synuclein proteins were removed and methylated with ^3^H-SAM using exogenous PIMT, and the level of isoaspartate present quantified by a methanol diffusion assay. Co-incubation of β-synuclein with α-synuclein resulted in a significant reduction in isoaspartate in native α-synuclein, and A53T mutant α-synuclein after 5 days, and after 2 days for A30P mutant α-synuclein - refer to Figure 7 left panels. This reduction of α-synuclein isoaspartate levels reached a significant 72.2±2.8%, 84.3±2.9%, and 47.9±3.74% (p<0.001) within native α-synuclein, A30P α-synuclein, and A53T α-synuclein respectively after 20 days of *in vitro* ageing – refer to Figure 7 left panels. This lowering of isoaspartate formation within the α-synucleins was also validated by methylation with ^3^H-SAM using exogenous PIMT, and gel separation of the ^3^H-methyl esters by 1D PAGE and their visualisation using autoradiography – Figure 7, right panels.

**Figure 8:**
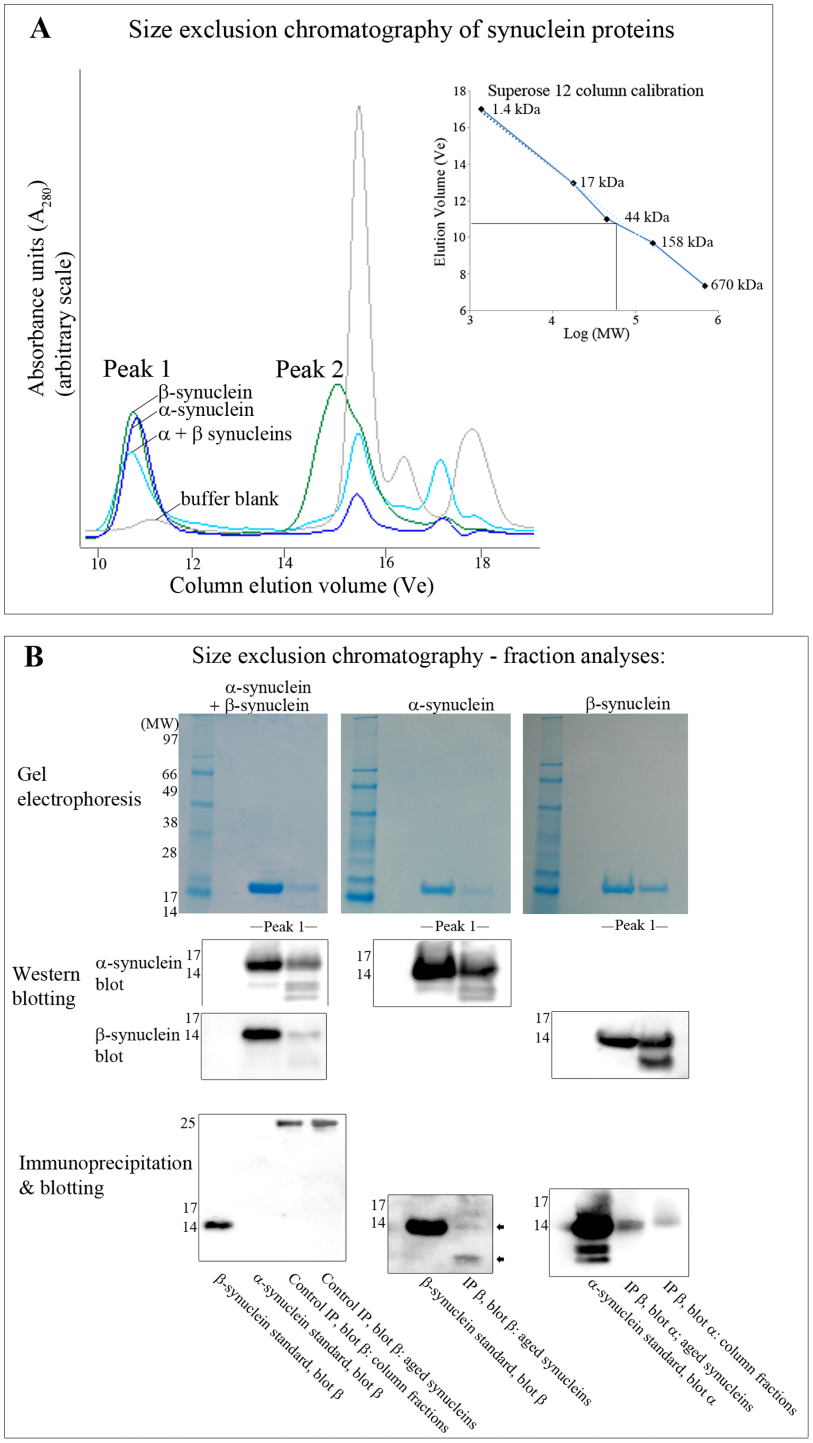
Size exclusion chromatography and immunoblotting of synuclein proteins. (**A**) Alpha, β, or co-incubated mixtures of both proteins were resolved by size exclusion chromatography on a Superose 12 column. Representative chromatograms from a buffer blank, and 20 day *in vitro* aged α-synuclein, β-synuclein, and co-incubation mixtures are shown. Synuclein proteins eluted as a single peak (Peak 1) in two column fractions at an elution volume of ∼10.89 ml, which corresponds to a molecular weight of ∼57.5 kDa. Peak 2 eluted at ∼15.20 ml, and was an artefact of the *in vitro* ageing buffer. The migration of proteins of known molecular weight was used to calibrate the column – see inset. (**B**) Peak column fractions were concentrated, resolved by 1D-PAGE, and stained with colloidal Coomassie blue (upper section), and Western blotted using specific anti-synuclein antibodies (middle section). Beta-synuclein was not immunoprecipitated from either column fractions or aged synuclein reactions when control serum was applied, and β-synuclein antibody did not cross-react with α-synuclein protein. Half of a µg of β-synuclein was similarly blotted to provide a protein standard (lower section, left panel). Beta-synuclein was immunoprecipitated directly from *in vitro* aged fractions, with the level of β-synuclein recovered confirmed by Western blotting. Parent β-synuclein protein as well as lower molecular weight proteolytic fragments were recovered (marked with arrowheads). Two and a half µg of β-synuclein was similarly blotted to provide a protein standard (lower section, central panel). Beta-synuclein immunoprecipitated from *in vitro* aged co-incubated mixtures, or from Superose 12 column peak fractions also detected the presence of α-synuclein by Western blotting. Two and half µg of α-synuclein was similarly blotted to provide a protein standard (lower section, right panel). The positions of protein molecular weight standards are also included on Western blots.

We predict that the known physical interaction of α-synuclein with β-synuclein [10]–[14], [43] can provide a mechanism for suppression of α-synuclein isoaspartate formation during co-incubation experiments. To assess this we resolved co-incubated α-synculein and β-synuclein by size exclusion chromatography in non-denaturing buffer, with results presented in Figure 8. Incubation of α- and β-synuclein with one another, or as individual proteins, resulted in the detection of two column peaks: Peak 1 eluted at ∼10.89 ml, and Peak 2 eluted at ∼15.20 ml. Peak 1 elution corresponded to a protein of molecular weight of ∼57.5 kDa, consistent with the formation of synuclein tetramers. Peak 2 was devoid of protein and was an artefact of the *in vitro* ageing buffer – Figure 8, Part A. Fractions from Peak 1 were concentrated and proteins resolved by denaturing 1D SDS-PAGE – Figure 8, Part B, upper panel. Co-incubation of α- and β-synucleins was recovered as a mixture of both proteins in a diffuse band of ∼14.5 kDa after Coomassie staining. Peak 1 fraction analysis by SDS-PAGE of α- or β-synucleins when run as single proteins also only recovered monomeric forms of the proteins. The identity of synuclein proteins was confirmed by Western blotting - Figure 8B middle panels.

The study of Israeli & Sharon (2009) has shown that native (*in vivo*) α- and β-synucleins can exist as hetero-oligomers [43]. However, for co-incubated synuclein proteins our size exclusion chromatography is unable to distinguish between the elution profile of homo-oligomers or hetero-oligomers. Thus to confirm molecular interaction of α- and β- synucleins we immunoprecipitated β-synuclein directly from *in vitro* aged co-incubated mixtures, or from Superose 12 column peak fractions from co-incubation analyses, and then blotted for α-synuclein. In either case, after immunoprecipitation with a β-synuclein specific antibody, α-synuclein was detected by subsequent Western blotting – Figure 8B, lower panels. The ability of the β-synuclein antibody to specifically immunoprecipitate β-synuclein was also confirmed, and was not achieved using control rabbit serum which was only immunoreactive against antibody chains (∼25 kDa light chain is shown). The β-synuclein antibody was also unreactive against α-synuclein (Figure 8B, lower panels).

## Discussion

The major recognised cellular function of PIMT is to limit the accumulation of detrimental isoaspartate peptide linkages that form within nuclear and cytosolic proteins during protein ageing, and as a consequence of cellular stresses [16]–[39]. Previously we pioneered the use of a proteomic approach to resolve and ultimately identify a number of the hitherto unknown murine targets of PIMT; and this included α-synuclein and β-synuclein [16]. Our 2D-PAGE approach was successful in separating the highly homologous murine α-synuclein and murine β-synuclein due to their differences in (theoretical) isoelectric point (pI): α-synuclein, pI 4.74, and β-synuclein pI 4.38 [16]. Human α-synuclein and β-synuclein possess high amino acid sequence homology to their murine counterparts (95 and 97% respectively), and also similar theoretical pIs: α-synuclein, pI 4.67, and β-synuclein, pI 4.41. Hence the human synuclein proteins were also able to be separated by 2D-PAGE, and migrated similarly to their murine versions when resolved by co-migrational analyses. Thus we assume that by similarity, these human synuclein proteins would be substrates of human PIMT, and be useful as models of accumulation of isoaspartate protein damage during *in vitro* protein ageing. Furthermore, we chose to examine the human proteins because of the known involvement of human α-synuclein in age-related pathologies (synucleopathies) [3], and also the availability of human synucleins as commercial, pure, recombinant proteins.

After *in vitro* ageing of α-synuclein at physiological pH and temperature for 20 days, 2D-PAGE resolved α-synuclein into two major Coomassie stained proteins, and β-synuclein to one broad Coomassie stained protein, and additional minor protein spots of both proteins. We assume these additional protein spots represent protein post-translational modifications, minor levels of proteolysis that occur during the *in vitro* ageing time course, and incomplete 2D-PAGE resolution. The presence of several similarly migrating forms of α- and β-synuclein would concur with 2D-PAGE separation of *Pcmt1−/−* knockout brain cytosolic proteins, which also detected a number of other isoaspartate containing minor protein spots juxtaposed to those identified as α-synuclein and β-synuclein, although their identity has yet to be confirmed [16].

Since the synuclein proteins were stable for up to 20 days of *in vitro* ageing (confirmed by Coomassie staining after 2D-PAGE) we quantified the level of isoaspartate protein damage that accumulated over that time course within these synucleins, and additionally extended the analysis to include the A30P and A53T mutants of α-synuclein. All α-synuclein proteins accumulated isoaspartate at a similar rate of ∼1% of molecules per day, suggesting that the presence of a pathological mutation in α-synuclein (A30P or A53T) does not greatly influence the initial rate of formation of isoaspartate. These rates are slower than that reported for the other PIMT *in vivo* substrates that have also been aged *in vitro*: synapsin I (MW ∼74 kDa) at ∼6%, and tubulin (MW ∼50 kDa) at ∼2% of molecules formed isoaspartate per day respectively [38], [39], but presumably this is a reflection of the smaller size of α-synucleins (MW ∼14 kDa), and hence formation of isoaspartate at fewer sites.

Noteworthy was that by comparison, the formation of isoaspartate within β-synuclein was approximately 20 times slower than that of the α-synucleins. These differences in rates and final levels of isoaspartate formation between these synucleins were further validated by exogenous isoaspartate methylation using PIMT, protein resolution by 1D PAGE, and visualisation of ^3^H-methyl esters by autoradiography. Silver staining of protein removed during the *in vitro* ageing time course supported the 2D-PAGE analysis and showed that the major parent protein remained intact for all synuclein forms, but a level of lower molecular weight proteins existed, consistent with minor proteolysis. In addition, stable α-synuclein oligomers that contained isoaspartate were also present for all α-synuclein subtypes, and these were resistant to the denaturing conditions of SDS-PAGE. The level of oligomers formed that were detected by protein staining increased with days of ageing. By comparison, stable higher molecular weight oligomers of β-synuclein were not seen during this ageing time course, although extension of β-synuclein *in vitro* ageing to 30 days did result in detection of oligomers (our unpublished observations). In support of our results, other independent studies have reported the ability of α-synuclein, and α-synuclein A30P and A53T mutants to readily form oligomers, but not β-synuclein [10]–[12], [14]. However, some of these studies have been undertaken with protein incubations at 65°C, which presumably accelerates the protein-protein interactions and oligomerisation of α-synucleins, enabling oligomerisation detection within hours rather than the days reported here for physiological ageing conditions.

A number of studies have also described the neuroprotective role of β-synuclein, suggesting that its interaction with α-synuclein may slow or limit the ability of α-synuclein to aggregate, form fibrils, and elicit a neurotoxic response [10]–[14], [43]. This led us to investigate if co-incubation of approximately equimolar β-synuclein with α-synuclein affected the formation of isoaspartate within α-synuclein, and also limited the formation of stable protein oligomers. Co-incubation of β-synuclein with α-synuclein significantly reduced total isoaspartate formation at 5, 9, and 20 day time points for all α-synuclein proteins. The mutant A30P α-synuclein was the most responsive protein, with a significant amelioration of isoaspartate protein damage detected after 2 days of co-incubation, and reduced by 84% from that of A30P α-synuclein alone after 20 days of ageing.

Stable protein oligomerisation of α-synucleins was not abolished under these conditions, with oligomers visible after autoradiography of radiolabelled isoaspartate methyl esters. Other studies have reported the ability of β-synuclein to significantly reduce oligomerisation of α-synuclein, although with some reports this has required a molar excess of β-synuclein rather than equimolar amounts of both proteins [10], [11]. Beta-synuclein may therefore be able to completely abolish both isoaspartate protein damage and protein oligomerisation within α-synucleins during this time course if a molar excess of β-synuclein was employed. However, our studies with cytosolic brain proteins from *Pcmt1+/+* (wild-type), or *Pcmt1−/−* mice suggest approximately similar α-synuclein and β-synuclein protein levels exist [16], and this has recently been supported by another group [43]. Hence we have restricted our analyses to near equimolar β-synuclein and α-synuclein co-incubations, although pathological situations may arise from an imbalance in the relative expression levels of α- to β-synuclein, or from β-synuclein mutations [44], [45].

A direct binding/association of β-synuclein to α-synuclein is the molecular mechanism reported by which β-synuclein is able to suppress α-synuclein aggregation, fibril formation, and neurotoxicity from *in vitro* and *in vivo* studies [10]–[14], [43]. Furthermore, studies have reported the presence of molecular interaction domains within β-synuclein that specifically bind α-synuclein, and these have been modelled using β-synuclein derived peptides [46].

Hence we similarly assumed that β-synuclein associates with α-synuclein and this sterically hinders the formation of isoaspartate within α-synuclein. To investigate this association, we resolved α- and β- co-incubated synucleins by non-denaturing size exclusion chromatography. Resolution of co-incubated proteins, or α- or β-synuclein alone, resulted in the detection of protein oligomers of ∼57.5 kDa; indicative of tetramers. However, this chromatography is not able to distinguish between the co-elution of homo-oligomers or hetero-oligomers. Hence we confirmed molecular interaction of α- and β-synucleins by their co-immunoprecipitation from either Superose 12 column peak fractions, or from *in vitro* aged co-incubated mixtures.

It will be of interest in future studies to ascertain which residues within human and murine α-synucleins and human and murine β-synucleins form isoaspartate peptide linkages, and how this post-translational modification influences synuclein protein properties. Human α-synuclein possesses 6 aspartic acid residues and 3 asparagine residues capable of giving rise to isoaspartate (residues highlighted in Figure 2). By comparison, there are only 3 aspartic acids and 1 asparagine in human β-synuclein, hence we might speculate that the higher rate and amount of isoaspartate that forms within α-synucleins arises from a greater number of isoaspartate forming sites than β-synuclein.

The incidence and rates of deamidation and isoaspartate formation have been examined using model peptides [47]–[49], and the half-life of deamidation of human α-synuclein has also recently been assessed [50]. These peptide model studies have suggested that isoaspartate forms more readily at sequences at which the side chain of the C-flanking amino acid is relatively small and hydrophilic, as opposed to C-flanking amino acids which are bulky or hydrophobic. Hence Asn-Gly, Asp-Gly, Asn-His, and Asn-Ser sequences when present within flexible regions of a protein displayed preference for isoaspartate formation via the succinimide intermediate [47]–[49]. Alpha-synuclein does exist in solution as a natively unfolded structure [51], a flexible conformation(s) that may facilitate isoaspartate formation. Furthermore, wild-type α-synuclein deamidation has been reported for Asn103 and Asn122, residues within the unfolded carboxyl region [50]. However, canonical isoaspartate hot-spot sequences are absent from either human α-synuclein or human β-synuclein, but the site specificity of isoaspartate formation and PIMT carboxyl methylation may yet be better evaluated in future studies using more proteins identified as *bona fide in vivo* substrates of PIMT [16], [52].

Although human and murine α-synucleins and β-synucleins have nearly identical amino acid sequences, subtle differences in their amino acid coding may result in profound changes in protein properties. For example, the amino acid change of A53 to T53 that can result in rare Parkinonian phenotypes in humans is the wild-type sequence in mice, but does not trigger a similar pathology. It is interesting to note that of the other 6 amino acid differences between the human α-synuclein and murine α-synuclein sequences, 4 of these are either an asparagine or asparatic acid residue, and therefore capable of forming isoaspartate peptide linkages. Whether rates and levels of isoaspartate differ greatly between human and murine α-synucleins will be evaluated in future studies. For β-synuclein, the four amino acids capable of forming isoaspartate are conserved between the two species, but notably, Asp121 of the human β-synuclein sequence is C-flanked by a proline residue, a sequence less likely to facilitate isoaspartate formation, whereas the murine sequence is C-flanked by a serine residue, in a sequence that would favour isoaspartate formation. Thus we would predict that murine β-synuclein would likely form isoaspartate at a higher rate and to a higher level than human β-synuclein, but this will also need to be evaluated with future studies. This supposition is in part supported by our study of *Pcmt1−/−* mice which had accumulated similar levels of isoaspartate in α- and β-synucleins (at the snap-shot analysis of ∼9 weeks of age) [16], suggesting that murine β-synuclein may form isoaspartate to a similar level to that of murine α-synuclein.

The functional consequences of isoaspartate protein damage in α-synuclein or β-synuclein will also warrant future study. Alpha and β-synucleins are co-localised presynaptic proteins and are thought to function in synaptic vesicle release and transmission, and neuronal plasticity [1]–[3]. *Pcmt1−/−* mice do display neurodegenerative damage with an extensive gliosis [27], [29], and exhibit aberrant presynaptic neurotransmission in the hippocampal CA3 region, learning deficits and impaired spatial memory as determined by a Morris water maze [29]. This indicates a role of PIMT and/or protein isoaspartate formation as key modulators of proteins involved in neurotransmission and neurodegeneration, but to assign a contribution of the *Pcmt1−/−* neurological deficits specifically to isoaspartate-damaged α-synuclein or β-synuclein is not feasible; since PIMT regulates isoaspartate levels in a number of other proteins known to be involved in these processes [16], [52].

It has been suggested for Aβ protein variants, (the primary peptide/protein constituents of brain amyloid fibrils in AD patients), that isoaspartate formation within Aβ may enhance the process of aggregation, and promote the conformational change that drives β-turn formation for generation of stable oligomeric β-sheet structures [24], [53]. This may enhance peptide stability, insolubility, and resistance to enzymatic degradation [53]–[55]. This will need to be evaluated for isoaspartate-rich forms of α-synuclein and β-synuclein, and how isoaspartate formation may also influence the repertoire of other synuclein post-translational modifications such as phosphorylation, ubiquitination, and protein truncation. Furthermore, these other post-translational modifications of α-synuclein may also be influenced by co-incubation with β-synuclein. Interestingly, the majority of truncated forms of α-synuclein terminate with either an aspartic acid (Asp115, Asp119, Asp135) or an asparagine residue (Asn122), which may arise from limited proteolysis [56]. Since isoaspartate peptide linkages are resistant to commonly encountered proteases [55], it remains to be determined whether examples of α-synuclein truncation *in vivo* arise from resistance to isoaspartate cleavage.

Finally, a comprehensive examination of the development of isoaspartate in α-synuclein within the proteinaceous aggregates that form in human synucleopathies will be of interest for future studies. Isoaspartate protein damage could accumulate within α-synuclein from sustained inactivation of PIMT, possibly as a consequence of a reduction in the ratio of SAM∶SAH [57], or simply as a consequence of protein ageing if α-synuclein is inaccessible to PIMT-mediated repair. Indeed, the inaccessibility of Aβ-peptide to PIMT repair may be the molecular mechanism responsible for the increased isoaspartate levels that accumulate in Aβ-peptide examined from AD brain tissue [24], [53], [54]. By either mechanism, an increase in the level of isoasparate within α-synuclein may have pathological consequences that include an increased propensity of protein aggregation and reduced protein clearance via proteolytic resistance.

In summary, human α-synucleins and human β-synuclein are proteins that are susceptible to a number of post-translational modifications, which includes the formation of isoaspartate protein damage. Since these two synuclein proteins are highly homologous, it is important to establish the differences in their molecular behaviour that may contribute to their differential propensity to contribute to synucleopathies. We have shown that β-synuclein is relatively resistant to isoaspartate formation and able to ameliorate its formation within α-synucleins, consistent with a neuroprotective action. It is provocative to consider that inhibition of methyltransferases such as PIMT, which may arise through lifestyle choices such as excessive alcohol consumption, could result in increased isoaspartate protein damage within PIMT substrates such as synucleins, and influence their (patho)physiological activities.

## Supporting Information

Supporting Information S1 **A sample Western blot of** ***in vitro*** **aged A53T mutant α-synuclein resolved by 1D PAGE (Figure S1), and a peptide mass fingerprint of the stable α-synuclein oligomers (Supplementary text), are both included as supporting data.**
(DOCX)
