# Supplementary material for: Molecular Ageing of Alpha- and Beta-Synucleins: Protein Damage and Repair Mechanisms
Source: PLoS One. 2013 Apr 22;8(4):e61442. doi: 10.1371/journal.pone.0061442 (PMC3632608; doi:10.1371/journal.pone.0061442)
Supplement: Supporting Information S1 — A sample Western blot of in vitro aged A53T mutant α-synuclein resolved by 1D PAGE (Figure S1), and a peptide mass fingerprint of the stable α-synuclein oligomers (Supplementary text), are both included as supporting data. (DOCX) [file pone.0061442.s001.docx]

**Supporting (Supplemental) Data:**

**MALDI-TOF mass spectrometry of α-synuclein oligomers.**

Silver stained protein bands from 1D PAGE gels of *in vitro* aged α-synuclein, or A30P, or A53T mutant α-synucleins were excised, digested with trypsin, and tryptic fragments mass analysed using MALDI-TOF mass spectrometry. An example of a peptide mass fingerprint from an α-synuclein oligomer is shown, with eight peptides identified (listed in bold) that corresponded to approximately 52 % protein coverage of human α-synuclein, and generated a MOWSE probability score of 109. A MOWSE protein score of greater than 83 was considered a significant match (*P* < 0.05) of tryptic peptides to those present within protein databases, to enable confident protein identification. The positions of the tryptic peptides detected within the human α-synuclein amino acid sequence are also shown.

St-End Observed Mr(expt) Mr(calc) Delta Miss Sequence

11 - 21 1072.5800 1071.5727 1071.5924 -0.0197 1 K.**AKEGVVAAAEK**.T

35 - 43 951.5000 950.4927 950.5073 -0.0146 0 K.**EGVLYVGSK**.T

35 - 45 1180.6200 1179.6127 1179.6499 -0.0372 1 K.**EGVLYVGSKTK**.E

46 - 58 1295.6700 1294.6627 1294.6881 -0.0254 0 K.**EGVVHGVATVAEK**.T

46 - 60 1524.8300 1523.8227 1523.8308 -0.0080 1 K.**EGVVHGVATVAEKTK**.E

59 - 80 2157.2200 2156.2127 2156.1801 0.0326 1 K.**TKEQVTNVGGAVVTGVTAVAQK**.T

61 - 80 1928.0800 1927.0727 1927.0375 0.0352 0 K.**EQVTNVGGAVVTGVTAVAQK**.T

81 - 96 1478.7900 1477.7827 1477.7777 0.0051 0 K.**TVEGAGSIAAATGFVK**.K

1 MDVFMKGLSK **AKEGVVAAAE K**TKQGVAEAA GKTK**EGVLYV GSKTKEGVVH**

51 **GVATVAEKTK EQVTNVGGAV VTGVTAVAQK TVEGAGSIAA ATGFVK**KDQL

101 GKNEEGAPQE GILEDMPVDP DNEAYEMPSE EGYQDYEPEA

**Figure S1**: **Western blot of *in vitro* aged A53T mutant α-synuclein resolved by 1D PAGE.** Positions of immune-reactive oligomers are marked with arrowheads.
